# Association of walking speed in late midlife with mortality: results from the Whitehall II cohort study

**DOI:** 10.1007/s11357-012-9387-9

**Published:** 2012-02-24

**Authors:** Alexis Elbaz, Séverine Sabia, Eric Brunner, Martin Shipley, Michael Marmot, Mika Kivimaki, Archana Singh-Manoux

**Affiliations:** 1 INSERM, U708, Neuroepidemiology, 75013 Paris, France; 2 UPMC Université Paris 06, UMR_S708, Neuroepidemiology, 75005 Paris, France; 3 INSERM, U1018, Centre for Research in Epidemiology and Population Health, Villejuif, France; 4 Université Paris 11, 94807 Villejuif, France; 5 Department of Epidemiology and Public Health, University College London, London, UK; 6 Centre de Gérontologie, Hôpital Ste Périne, AP-HP, Paris, France; 7 INSERM, U708, Hôpital de la Salpêtrière, 47 boulevard de l’Hôpital, 75651 Paris Cedex 13, France

**Keywords:** Walking speed, Mortality, Inflammation, Cohort study, Epidemiology

## Abstract

Slow walking speed is associated with increased mortality in the elderly, but it is unknown whether a similar association is present in late midlife. Our aim was to examine walking speed in late midlife as a predictor of mortality, as well as factors that may explain this association. Data are drawn from the Whitehall II longitudinal cohort study of British civil servants. The analyses are based on 6,266 participants (29% women; mean age = 61 years, SD = 6) for whom “walking speed at usual pace” was measured over 8 ft (2.44 m) at baseline. Participants were followed for all-cause and cause-specific mortalities during a mean of 6.4 (SD = 0.8) years. During this period, 227 participants died. Participants in the bottom sex-specific third of walking speed (men, <1.26 m/s; women, <1.09 m/s) had an increased risk of death compared to those in the middle and top thirds (age- and sex-adjusted hazard ratio = 1.89, 95% confidence interval (CI) = 1.45–2.46), with no evidence of effect modification by age or sex (interactions, *P* ≥ 0.40). The association between walking speed and mortality was partially explained by baseline inflammatory markers (percentage reduction of the association 22.8%), height and body mass index (16.6%), chronic diseases (14.0%), and health behaviors (13.4%). Together these and other baseline factors (socioeconomic status, cardiovascular risk factors, cognitive function) explained 48.5% of the association (adjusted hazard ratio = 1.39, 95% CI = 1.04–1.84). In conclusion, walking speed measured in late midlife seems to be an important marker of mortality risk; multiple factors, in particular inflammatory markers, partially explain this association.

**Electronic supplementary material:**

The online version of this article (doi:10.1007/s11357-012-9387-9) contains supplementary material, which is available to authorized users.

## Introduction

Lower walking speed in the elderly has been associated with increased all-cause (Cooper et al. 2010) and cardiovascular mortalities (Dumurgier et al. 2009). In a recent pooled analysis of nine US cohorts, the hazard ratio for death per 0.1-m/s increase in walking speed was 0.88 (95% confidence interval (CI) = 0.87–0.90). The authors concluded that walking speed may be a simple and accessible indicator of the health of elderly people (Studenski et al. 2011); mean age of participants at baseline in these studies varied between 72 and 79 years. Another recent meta-analysis highlighted the lack of studies that measured walking speed in younger populations with subsequent follow-up for mortality (Cooper et al. 2010). It is therefore unknown whether walking speed at earlier ages is associated with mortality—as it is in older age groups. Moreover, very few studies have examined factors that may explain the association between slower walking speed and mortality. Elucidating pathways that link worse physical performances to mortality may help to define potential new intervention strategies (Cooper et al. 2010).

The objectives of the current investigation were twofold. First, using the data from the Whitehall II cohort study, we examined whether walking speed measured in late midlife is associated with mortality. Second, in order to improve understanding of the potential underlying pathways linking walking speed and mortality, we assessed the contributions of a wide range of covariates, including sociodemographic and clinical factors, chronic conditions, cognition, health behaviors, and inflammatory markers.

## Methods

### Participants

The Whitehall II cohort study, established in 1985, is a longitudinal study of 10,308 civil servants (Marmot et al. 1991). All civil servants aged 35–55 years in 20 London-based departments were invited to participate (participation rate, 73%). The baseline examination (phase 1, 1985–1988) included a clinical examination and a self-administered questionnaire. Subsequent phases have alternated between postal questionnaires alone (phases 2 (1988–90), 4 (1995–6), 6 (2001), and 8 (2006)) or accompanied by a clinical examination (phases 3 (1991–3), 5 (1997–9), 7 (2002–4), and 9 (2007–9)). Participants gave a written consent, and the study was approved by the University College London Ethics Committee.

### Walking speed

Walking speed was measured at phase 7 (2002–4) over a clearly marked 8-ft (2.44 m) walking course using a standardized protocol (Brunner et al. 2009). Participants were asked to “walk to the other end of the course at your usual walking pace, just as if you were walking down the street to go the shops. Walk all the way past the other end of the tape before you stop.” The starting position was standing at the start of the course. A trained nurse walked behind the participant and stopped timing when the participant’s foot hits the floor after the end of the walking course. Three tests were conducted, and the fastest measure was used in the analysis.

### Mortality

A total of 10,297 respondents (99.9%) were traced for mortality through the national mortality register (National Health Services Central Registry) using the National Health Service identification number assigned to British citizens. Our analyses are based on deaths that occurred between phase 7 (2002–4) and January 31st, 2010. We examined all-cause, cardiovascular, and cancer mortalities; international classification of disease (ICD) codes identified cancer (ICD-9, 140.0–209.9; ICD-10, C00–C97) and cardiovascular (ICD-9, 390.0–458.9; ICD-10, I00–I99) deaths.

### Covariates

*Sociodemographic variables* included age, sex, and socioeconomic status (SES) (based on 6-level British civil service employment grade at phase 7, which is related to salary, social status, and level of responsibility (Marmot et al. 1991).

*Health behaviors* were drawn from questionnaires (phases 1, 3, 5, and 7). *Smoking history* was assessed using questions on smoking status and current amount of tobacco smoked categorized as “current smoker at phase 7,” “recent ex-smoker” (stopped smoking between phases 1–7), “long-term ex-smoker” (stopped smoking before phase 1), and “never smoker.” *Alcohol consumption* was assessed via questions on the number of alcoholic drinks (“measures” of spirits, “glasses” of wine, and “pints” of beer) consumed in the last 7 days at each phase. This was converted to number of units (1 unit = 8 g) of alcohol. The *frequency of fruit* *and vegetable* *consumption* was assessed on an eight-point scale (ranging from “seldom or never” to “two or more times a day”). The *number of* *hours of* *moderate and* *vigorous physical* *activity per* *week* was calculated from a question on the number of hours of physical activity at different levels (phases 1 and 3) and from a 20-item questionnaire on frequency and duration of participation in walking, cycling, sports, gardening, housework, and home maintenance (phases 5 and 7) (Sabia et al. 2009). Summary measures of health behaviors over the four phases were used in the analyses: smoking history as defined above and mean values over four phases for other health behaviors.

*Body mass* *index* (BMI) was calculated as weight divided by height squared (kg/m²) measured at phase 7 clinical examination (Sabia et al. 2010), and categorized as <20, 20.0–24.9, 25–29.9, and ≥30 kg/m^2^.

*Cardiovascular risk* *factors* included serum total cholesterol and systolic/diastolic blood pressure (phases 1, 3, 5, and 7) (Sabia et al. 2010). The mean of four measures (phases 1, 3, 5, and 7) reflected the history of these risk factors in the analysis. Heart rate (bpm, beats/minute) was measured at phase 7 via an electrocardiogram on participants in the supine position and was categorized for the analysis (<60, 60–80, and >80 bpm) (Fuster et al. 2001).

*Chronic conditions* included prevalence until phase 7 of coronary heart disease (CHD), stroke, arthritis, respiratory illness, diabetes, and depression. CHD (myocardial infarction, definite angina) was based on clinically verified events (Ferrie et al. 2006). Stroke, arthritis, and respiratory illness were assessed through self-reports. Diabetes was defined by a fasting glucose ≥7.0 mmol/L or a 2-h postload glucose ≥11.1 mmol/L or a reported doctor-diagnosed diabetes, or by the use of diabetes medication (Expert committee on the diagnosis and classification of diabetes mellitus 2003). Self-report on the use of antidepressant drugs in the 14 days preceding phase 7 was included as an indicator of current depression; depressive symptoms in the last 7 days at phase 7 were assessed using the Center for Epidemiologic Studies Depression (CES-D) scale. Use of NSAIDs was assessed in a similar manner. Forced vital capacity and FEV_1_ were measured at phase 7 using a portable flow spirometer in a subset of participants (Sabia et al. 2010).

*Inflammatory markers* (interleukin-6, IL-6; C-reactive protein, CRP) were measured at phases 3 and 7 (Sabia et al. 2010; Elovainio et al. 2010). As their distributions were skewed, they were log-transformed for the analysis, and the mean of two measures were used in the analysis.

*Cognitive status* was assessed at phase 7 using a test of reasoning (*Alice Heim* *4-I*, *AH4-I*); higher scores correspond to better function (Heim 1970).

### Statistical analysis

Analyses were undertaken using STATA 10 (StataCorp LP, TX, USA) and SAS 9.1 (SAS Institute, NC, USA). Descriptive statistics were used to examine participants’ characteristics as a function of vital status and tertiles of walking speed. As men walked faster than women, walking speed was categorized using sex-specific tertiles, and these groups were compared using linear or logistic regression with adjustment for age and sex.

For the survival analyses, participants were followed-up until death or until the date of censoring for those who were alive (date of emigration or January 31st, 2010), whichever occurred first. The cumulative risk of all-cause death was plotted by tertiles of walking speed which are defined using sex-specific cutoffs (men <1.26, 1.26–1.45, and >1.45 m/s; women <1.09, 1.09–1.30, and >1.30 m/s), and the logrank test was used for statistical testing. Cox proportional hazards models with age as the time axis estimated hazard ratios (HR) and 95% CI. The proportional hazards assumption was verified using Schoenfeld residuals. Interactions were tested by including multiplicative terms in the models.

Model 1 included walking speed and sex as predictors and was controlled for age by considering it as the timescale. We examined the extent to which the association between walking speed and mortality was attenuated by adding the following covariates (models 2–8): SES; height and BMI, health behaviors, cardiovascular risk factors, chronic conditions, cognitive function, and inflammatory markers. The final model (model 9) included all covariates. The percentage reduction (PR) of the association between walking speed and mortality attributed to covariates included in model *i* was calculated using the formula 100 × (*β*_Model 1_ − *β*_Model *i*_) / (*β*_Model 1_), where *β* is the coefficient obtained from the Cox proportional hazards model.

## Results

The analyses were based on 6,266 participants, with complete data on walking speed and covariates, contributing to a total of 39,892 person-years. Compared to the baseline of the Whitehall II study (phase 1, 1985–8), those included in the analysis were younger, more likely to be men, and were from the higher SES group (*P* < 0.001). Two hundred seventeen participants examined at phase 7 were not included in the analysis due to missing walking speed data: these persons were older (62.0 [SD = 6.1] vs 61.1 [SD = 6.0] years, *P* = 0.02), more likely to be women (34.6% vs 29.0%, *P* = 0.08), and were from the lower SES group (28.6% vs 23.7%, *P* = 0.10) than those who were included. Excluded participants had higher mortality (6.0% vs 3.6%), but this difference was not statistically significant after adjustment for age, sex, and SES (*P* = 0.15).

During a mean follow-up of 6.4 (SD = 0.8, range = 0.2–7.3) years, 227 of the 6,266 participants included in the analyses died (mortality rate = 5.7/1,000 person-years): 111 from cancer (mortality rate = 2.8/1,000 person-years) and 62 from cardiovascular disease (mortality rate = 1.6/1,000 person-years). Table 1 summarizes participants’ characteristics at the start of the follow-up as a function of vital status at the end of follow-up. In age- and sex-adjusted analyses, those who died were older, more likely to be smokers, consumed more alcohol, exercised less, and had a higher BMI, heart rate, and levels of inflammatory markers. They also had a lower cognitive score, walking speed, and cholesterol level.

**Table 1:** Characteristics of the study population according to vital status at the end of the follow-up

Characteristics		Alive at the end of follow-up (*N* = 6039)	Dead at the end of follow-up (*N* = 227)	*P* value^a^
Sociodemographic factors				
Age	*M* (SD)	60.9 (5.9)	64.3 (6.1)	<0.001
Women	*N* (%)	1746 (28.9)	71 (31.3)	0.60
Low socioeconomic status	*N* (%)	1413 (23.4)	70 (30.4)	0.12
Health behaviors				
Current smokers	*N* (%)	478 (7.9)	28 (12.3)	0.004
Units of alcohol/week	*M* (SD)	11.7 (11.7)	12.9 (14.6)	0.008
Frequency of fruit and vegetable consumption/week	*M* (SD)	8.0 (3.3)	7.8 (3.4)	0.14
Hours of moderate and vigorous physical activity/week	*M* (SD)	3.7 (2.7)	3.0 (2.4)	<0.001
Height (cm)	*M* (SD)	171.1 (9.3)	169.1 (9.8)	0.02
BMI (kg/m²)	*M* (SD)	26.7 (4.3)	28.2 (5.2)	<0.001
Cardiovascular risk factors				
Systolic blood pressure (mmHg)	*M* (SD)	123.4 (12.3)	126.2 (12.6)	0.11
Diastolic blood pressure (mmHg)	*M* (SD)	76.9 (7.9)	77.6 (8.7)	0.50
Total blood cholesterol (mmol/L)	*M* (SD)	6.0 (0.9)	5.9 (1.0)	0.03
Heart rate (beats/min)	*M* (SD)	68.1 (12.1)	70.3 (13.0)	0.009
Chronic conditions				
Diabetes	*N* (%)	573 (9.5)	45 (19.8)	<0.001
Coronary heart disease	*N* (%)	540 (8.9)	45 (19.8)	<0.001
Stroke	*N* (%)	146 (2.4)	16 (7.1)	0.003
History of arthritis	*N* (%)	1569 (26.0)	79 (34.8)	0.22
History of respiratory disease	*N* (%)	802 (13.3)	34 (15.0)	0.43
Anti-depressant use	*N* (%)	208 (3.4)	11 (4.9)	0.20
Cognitive function				
AH4-I score	*M* (SD)	43.7 (11.3)	40.1 (12.5)	0.01
Inflammatory markers				
CRP (log transformed) (mg/L)	*M* (SD)	0.07 (1.00)	0.54 (1.04)	<0.001
IL-6 (log transformed) (pg/mL)	*M* (SD)	0.53 (0.54)	0.81 (0.61)	<0.001
Walking speed (m/s)	*M* (SD)	1.3 (0.3)	1.2 (0.3)	<0.001

End of follow-up was defined as January 31st, 2010 or date of censoring, whichever occurred first

*M* mean, *SD* standard deviation

^a^Calculated using analysis of covariance for continuous variables and logistic regression for categorical variables; *P* values are adjusted for age and sex

**Fig. 1:**
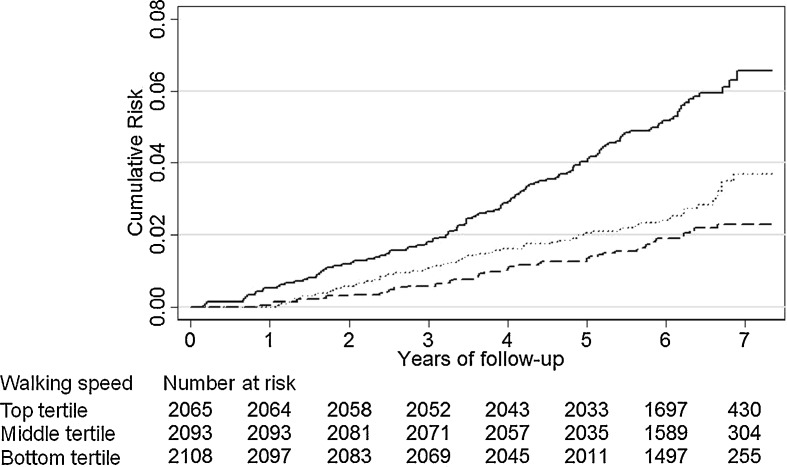
Cumulative risk of all-cause death according to sex-specific tertiles of walking speed (*dashed line*, *top*; *dotted line*, *middle*; *solid line*, *bottom*) (logrank, *P* < 0.001). The sex-specific cutoffs were <1.26 (*N* = 1,461), 1.26–1.45 (*N* = 1,520), and >1.45 m/s (*N* = 1,468) in men; <1.09 (*N* = 604), 1.09–1.30 (*N* = 573), and >1.30 m/s (*N* = 640) in women

Table 2 shows participants’ characteristics by tertiles of walking speed. Mean (SD) walking speed was higher in men (1.4 [0.3]) than women (1.2 [0.3], *P* < 0.001). There was a significant linear association between all other covariates and walking speed, except for alcohol consumption and history of respiratory disease. Subjects who walked slower had higher levels of inflammatory markers (*P* < 0.001). As shown in Fig. 1, there was also a statistically significant difference in the cumulative risk of all-cause mortality across tertiles of walking speed (logrank, *P* < 0.001). The curves began to separate during the first year of follow-up and continued to diverge over the follow-up. Differences in cumulative risk of death were also evident for cardiovascular (*P* < 0.001) and cancer (*P* < 0.001) deaths (results not shown).

**Table 2:** Characteristics of the study population according to tertiles of walking speed

Characteristics		Sex-specific tertiles of walking speed^a^			*P* value^b^
		Top (*N* = 2,065)	Middle (*N* = 2,093)	Bottom (*N* = 2,108)	
Sociodemographic factors					
Age	*M* (SD)	59.8 (5.6)	60.7 (5.9)	62.7 (6.0)	<0.001
Women	*N* (%)	604 (29.3)	573 (27.4)	640 (30.4)	<0.001
Low socioeconomic status	*N* (%)	330 (16.0)	449 (21.5)	704 (33.4)	<0.001
Health behaviors					
Current smokers	*N* (%)	127 (6.2)	173 (8.3)	206 (9.8)	<0.001
Units of alcohol/week	*M* (SD)	11.9 (11.5)	12.0 (11.7)	11.2 (12.4)	0.18
Frequency of fruit and vegetable consumption/week	*M* (SD)	8.6 (3.2)	8.0 (3.2)	7.4 (3.2)	<0.001
Hours of moderate and vigorous physical activity/week	*M* (SD)	3.8 (2.6)	3.8 (2.7)	3.4 (2.6)	<0.001
Height (cm)	*M* (SD)	172.0 (8.7)	171.6 (9.3)	169.6 (9.6)	<0.001
BMI (kg/m²)	*M* (SD)	26.0 (3.9)	26.5 (4.1)	27.7 (4.9)	<0.001
Cardiovascular risk factors					
Systolic blood pressure (mmHg)	*M* (SD)	122.2 (12.3)	123.3 (12.1)	124.9 (12.4)	<0.001
Diastolic blood pressure (mmHg)	*M* (SD)	76.3 (8.0)	76.8 (7.9)	77.5 (7.8)	<0.001
Total blood cholesterol (mmol/L)	*M* (SD)	5.9 (0.9)	6.0 (0.9)	6.1 (0.9)	0.05
Heart rate (beats/min)	*M* (SD)	67.5 (11.9)	68.6 (12.2)	68.4 (12.4)	<0.001
Chronic conditions					
Diabetes	*N* (%)	126 (6.1)	197 (9.4)	295 (14.0)	<0.001
Coronary heart disease	*N* (%)	143 (6.9)	174 (8.3)	268 (12.7)	<0.001
Stroke	*N* (%)	39 (1.9)	38 (1.8)	85 (4.0)	0.002
History of arthritis	*N* (%)	461 (22.3)	486 (23.2)	701 (33.3)	<0.001
History of respiratory disease	*N* (%)	276 (13.4)	276 (13.2)	284 (13.5)	0.23
Antidepressant use	*N* (%)	55 (2.6)	69 (3.3)	95 (4.6)	<0.001
Cognitive function					
AH4-I score	*M* (SD)	46.4 (9.8)	44.3 (10.9)	39.9 (12.3)	<0.001
Inflammatory markers					
CRP (log transformed) (mg/L)	*M* (SD)	−0.09 (0.99)	0.04 (0.98)	0.32 (1.00)	<0.001
IL-6 (log transformed) (pg/mL)	*M* (SD)	0.45 (0.52)	0.51 (0.53)	0.65 (0.58)	<0.001
Death at the end of the follow-up	*N* (%)	44 (2.1)	62 (3.0)	121 (5.7)	<0.001

*M* mean, *SD* standard deviation

^a^Based on cutoffs defined by tertiles of the distribution of walking speed in men and women: <1.26, 1.26–1.45, and >1.45 m/s in men; <1.09, 1.09–1.30, and >1.30 m/s in women

^b^Percentages of categorical variables and means of continuous variables are presented by tertiles of walking speed for clarity; walking speed was considered as the dependent variable in linear regression models to compute *P* values. *P* values are adjusted for age and sex

Cox proportional hazards model adjusted for age and sex with participants in the top tertile of walking speed as the reference (men, >1.45 m/s; women, >1.30 m/s) showed the HR of death to be 1.30 (95% CI = 0.89–1.92, *P* = 0.18) for participants in the middle tertile (men, 1.26–1.45 m/s; women, 1.09–1.30 m/s) and 2.19 (95% CI = 1.54–3.11, *P* < 0.001) for participants in the bottom (men, <1.26 m/s; women, <1.09 m/s). Because there was no statistically significant difference between the top and middle tertiles of walking speed for all-cause (as well as for cardiovascular and cancer mortalities), further analyses combined these two groups. In sensitivity analyses, we also modelled walking speed as a continuous variable (Table S1).

In Table 3, model 1 shows that participants in the lowest tertile of walking speed had a higher risk of death (HR = 1.89 [1.45–2.46]) compared to participants who walked faster. This association was not modified by age: the HR was 2.17 (1.04–4.50) for subjects in the lowest quartile of age (≤56.0 years; mean age = 53.9, range = 50.5–56.0 years) and 2.09 (1.41–3.09) for those in the highest quartile (>66.2 years; mean age = 69.2, range = 66.3–73.9 years) (interaction, *P* = 0.58). The association between walking speed and mortality was stronger in men (HR = 2.00 [1.46–2.75]) than women (HR = 1.68 [1.04–2.71]), but the difference was not statistically significant (*P* = 0.40); therefore, all analyses combine men and women and are adjusted for sex and age. SES (model 2), cardiovascular risk factors (model 5), and cognitive function (model 7) explained a small part of the walking speed–mortality association (PR = 3.3–6.4%). Adjustment for height and BMI (model 3), health behaviors (model 4), and chronic conditions (model 6) led to PRs of a similar magnitude (13.4–16.6%). The highest PR (22.8%) was on adjustment for inflammatory markers (model 8). All the covariates together explained nearly half (PR = 48.5%) the association; in the fully adjusted model, higher IL-6 (*P* = 0.04) and CRP (*P* = 0.04) remained associated with mortality. Analyses based on continuous walking speed yielded similar findings (Table S1).

**Table 3:** Hazard ratios for all-cause mortality according to walking speed (bottom tertile vs middle and top tertiles combined)

	All-cause mortality		
*N* deaths / *N*	227/6,266		
Adjustment	HR^a^	95% CI	%∆
Model 1: adjusted for age and sex	1.89	1.45–2.46	–
Model 2: model 1 + socioeconomic status	1.85	1.41–2.42	3.3
Model 3: model 1 + height + BMI	1.70	1.29–2.23	16.6
Model 4: model 1 + health behaviors^b^	1.73	1.32–2.27	13.4
Model 5: model 1 + cardiovascular risk factors^c^	1.86	1.42–2.42	2.7
Model 6: model 1 + chronic conditions^d^	1.73	1.32–2.26	14.0
Model 7: model 1 + cognitive function^e^	1.81	1.38–2.38	6.4
Model 8: model 1 + inflammatory markers^f^	1.63	1.25–2.14	22.8
Model 9: fully adjusted	1.39	1.04–1.84	48.5

^a^HR computed using Cox proportional hazards models with age as the time axis for participants in the bottom tertile of walking speed (<1.26 m/s in men, <1.09 m/s in women) compared to participants in the top and middle tertiles combined. The assumption of proportionality of hazards was verified (*P* = 0.39). The percentage reduction (%∆) of the association between walking speed and mortality attributed to covariates included in model *i* was calculated using the formula 100 × (*β*_Model 1_ − *β*_Model *i*_) / (*β*_Model 1_), where *β* is the regression coefficient obtained from the Cox proportional hazards model

^b^Smoking history, alcohol consumption, physical activity, and fruit and vegetable consumption

^c^Systolic and diastolic blood pressure, blood cholesterol, and heart rate

^d^History of diabetes, coronary heart disease, self-reported stroke, arthritis, respiratory diseases, and antidepressant use at phase 7

^e^AH4-I test

^f^Interleukin-6 and C-reactive protein (log-transformed)

Analyses for the two main causes of death (cardiovascular, cancer) are shown as supplementary results (Table S2). The HR was higher for cardiovascular (HR = 2.24 [1.34–3.74]) than for cancer (HR = 1.62 [1.11–2.36]) mortality; these relationships were not modified by age (cardiovascular, *P* = 0.54; cancer, *P* = 0.99) or sex (cardiovascular, *P* = 0.46; cancer, *P* = 0.75). For both causes, adjustment for inflammatory markers led to an important reduction in HRs (cardiovascular, PR = 23.4%; cancer, PR = 21.2), and all the covariates explained nearly half of the association (cardiovascular, PR = 49.3%; cancer, PR = 48.2%). Analyses based on continuous walking speed yielded similar findings (Table S1).

In sensitivity analyses, we excluded deaths occurring within the first 2 years of follow-up (*N* = 44); the association between walking speed and mortality remained unchanged for all-cause (HR = 1.80 [1.34–2.42]). Cardiovascular (HR = 1.99 [1.13–3.51]) and cancer mortalities (HR = 1.51 [0.98–2.31) in analyses were adjusted for age and sex. When we excluded subjects with a history of CHD, stroke, or diabetes at baseline (*N* = 1,194), the age and sex adjusted HR for all-cause (1.76 [1.26–2.45]) and cardiovascular mortalities (1.85 [0.90–3.79]) decreased, but adjustment for inflammatory markers still led to an important attenuation of the association (all-cause, PR = 25.3%; cardiovascular, PR = 29.9%). Using CES-D score rather than antidepressant use as a surrogate for depression, adjustment for forced expiratory volume/height² instead of history of respiratory disease, exclusion of NSAIDs users (*N* = 320), or participants with CRP level greater than 10 g/L (*N* = 103) on the day of examination led to conclusions similar to those in main analyses.

## Discussion

In the Whitehall II cohort study, walking speed was assessed in late midlife, and persons who walked slower had an increased risk of dying during a mean follow-up of 6.4 years. This relationship was also observed for the two main causes of death (cardiovascular and cancer) and was present in all age groups. Among a large number of covariates, inflammatory markers, and, to a lesser extent, chronic diseases, health behaviors, and height and BMI contributed to this association.

Several studies have shown that slower walking speed is associated with increased mortality (Cooper et al. 2010; Studenski et al. 2011). However, all of them measured walking speed in elderly subjects. To our knowledge, this is the first study to have examined whether this association is present when walking speed is measured in late midlife. We measured walking speed at a median age of 60 years, i.e., over 10 years earlier than previous studies (Cooper et al. 2010). In a recent pooled analysis of nine US cohorts, the mean age of the participants was comprised between 71.8 and 78.9 years; baseline walking speed was comprised between 0.56 and 1.19 m/s and was therefore markedly slower than in the present study (Studenski et al. 2011). Although participants from our study were younger than those from previous studies and thus walked faster, the HR for death per 0.1-m/s increase in walking speed was remarkably similar in the pooled analysis (HR = 0.88) and in our study (HR = 0.87). These findings suggest that walking speed can also be considered as a simple and accessible indicator of health in late midlife.

The factors that statistically explain the association between walking speed and mortality include confounders, common causes, and mediators and are likely to be complex. We examined the role of covariates primarily to highlight their role as “common causes.” A previous study, in which walking speed was measured in subjects aged 65–85 years, reported that walking speed was more strongly associated with cardiovascular than cancer deaths, which suggests that cardiovascular risk factors and diseases play a role (Dumurgier et al. 2009). In agreement with this hypothesis, we found that diabetes, stroke, and CHD—which were associated with slower walking speed and mortality—attenuated the association, in particular for cardiovascular mortality. On the other hand, cardiovascular risk factors, such as blood pressure, cholesterol, and heart rate made a modest contribution.

The most important contributing factors to the association between walking speed and mortality were inflammatory markers; their role was also apparent after excluding participants with stroke, CHD, or diabetes. Elevated inflammatory markers are associated with a wide range of conditions, including clinical and subclinical cardiovascular disease (Ridker et al. 2000a; Ridker et al. 2000b; Rodondi et al. 2010), diabetes, pulmonary disease, and chronic kidney disease, many of which are mortality risk factors (Chang et al. 2010). There is also an evidence to suggest that chronic inflammation represents a feature of the aging process itself (Franceschi et al. 2007). Inflammatory markers are associated in elderly subjects with a wide range of outcomes, including disability (Kuo et al. 2006), worse motor performances (Hsu et al. 2009), frailty (Leng et al. 2007), and death (Cappola et al. 2003). Our study extends this evidence by showing (1) that inflammatory markers are cross-sectionally associated with worse physical performance in late midlife and (2) that inflammatory markers explain a significant part of the association between walking speed and mortality, even in the absence of overt cardiovascular disease. Inflammatory markers may reflect subclinical cerebrovascular disease (Rosenberg 2009; Yoshida et al. 2009; Yoshida et al. 2010), which is associated with worse clinical performances (Soumaré et al. 2009) and mortality (Bokura et al. 2006; Ikram et al. 2009). Decreased muscle strength has been linked to inflammation (Degens 2010) and mortality (Landi et al. 2010; Newman et al. 2006) and may represent another possible pathway. Our findings suggest that inflammatory mechanisms associated with adverse outcomes in elderly subjects may be at play many years earlier; this observation has important implications because it may help to better define the time period that should be the target of preventive interventions.

Persons who walked slower had an increased risk of dying from cancer in our study, but a corresponding association has not been observed in a previous study on elderly persons (Dumurgier et al. 2009). Reasons for this discrepancy are unclear but may be related to differences in the types of cancer or age of the participants. In the present study, health behaviors played a stronger role than chronic conditions in explaining the association. Inflammatory markers also attenuated the association between walking speed and cancer mortality; however, they did not remain associated with cancer mortality in the fully adjusted model, thus suggesting that their effect was explained by other covariates.

Main strengths of this study include a standardized objective measure of motor performances in late midlife and the assessment of a wide range of covariates over a 15-year period preceding the walking speed measure. Short-distance walking speed has been measured in different studies over different distances (usually comprised between 2 and 10 m) (Graham et al. 2008; Studenski et al. 2011); we measured walking speed over 8 ft, but it has been shown that these measures are highly correlated and predict mortality in a similar way (Studenski et al. 2011). A limitation of our study is that white-collar civil servants are not representative of the general population; the sample, however, covered a wide socioeconomic range (Marmot et al. 1991). As our analyses are based on participants who took part in phase 7 of a longitudinal study, we did not include subjects who died earlier and a small number of subjects for whom walking speed was not measured. Finally, for cause-specific mortality, our analyses are based on small numbers of events.

Despite these limitations, our findings provide a strong evidence to suggest that slow walking speed during late midlife is associated with increased mortality over 6 years of follow-up and thus may be a good marker of general health in late midlife. Our results also emphasize the importance of inflammatory markers in explaining this association.

## Electronic supplementary material

Below is the link to the electronic supplementary material.

ESM 1 (PDF 80 kb)
