# Supplementary material for: Association of walking speed in late midlife with mortality: results from the Whitehall II cohort study
Source: Age (Dordr). 2012 Feb 24;35(3):943–52. doi: 10.1007/s11357-012-9387-9 (PMC3636402; doi:10.1007/s11357-012-9387-9)
Supplement: Supplementary file 1 — (PDF 80 kb) [file 11357_2012_9387_MOESM1_ESM.pdf]

Table S1 *Hazard ratios for all-cause, cardiovascular, and cancer mortality per 0.1 m/s higher walking speed (continuous variable).*

	**All-cause mortality**			**Cardiovascular mortality**			**Cancer mortality**		
**N deaths / N**	**227/6266**			**62/6266**			**111/6266**		
**Adjustment**	**HR** [a]	**95% CI**	**% Δ**	**HR** [a]	**95% CI**	**% Δ**	**HR** [a]	**95% CI**	**% Δ**
Model 1: Adjusted for age and sex	0.87	0.83-0.92	--	0.86	0.79-0.94	--	0.89	0.84-0.96	--
Model 2: Model 1 + socioeconomic status	0.88	0.83-0.92	1.4	0.87	0.79-0.95	5.7	0.89	0.83-0.95	-8.0
Model 3: Model 1 + height + BMI	0.89	0.85-0.94	15.3	0.88	0.80-0.96	14.1	0.91	0.85-0.97	13.8
Model 4: Model 1 + health behaviours [b]	0.89	0.85-0.93	11.6	0.88	0.80-0.96	13.3	0.91	0.85-0.97	12.5
Model 5: Model 1 + cardiovascular risk factors [c]	0.88	0.84-0.92	4.3	0.87	0.79-0.95	4.4	0.90	0.84-0.96	5.1
Model 6: Model 1 + chronic conditions [d]	0.89	0.85-0.93	14.8	0.89	0.82-0.98	25.7	0.91	0.85-0.97	15.6
Model 7: Model 1 + cognitive function [e]	0.88	0.84-0.92	4.6	0.87	0.79-0.95	6.8	0.89	0.83-0.96	-1.5
Model 8: Model 1 + inflammatory markers [f]	0.90	0.86-0.94	20.5	0.89	0.81-0.98	23.7	0.91	0.85-0.98	17.1
Model 9: Fully-adjusted	0.93	0.88-0.98	44.3	0.94	0.85-1.03	55.6	0.93	0.86-1.00	35.8

[a] HR computed using Cox proportional-hazards models with age as the time-axis for an increase of 0.1 m/s in walking speed. The assumption of proportionality of hazards was verified (all-cause, P=0.41; cardiovascular, P=0.85; cancer, P=0.25). The percentage reduction (% Δ) of the association between walking speed and mortality attributed to covariates included in model *i* was calculated using the formula 100 x ($\beta_{Model\ 1}$ - $\beta_{Model\ i}$)/($\beta_{Model\ 1}$), where β is the regression coefficient obtained from the Cox proportional hazards model.
[b] Smoking history, alcohol consumption, physical activity, and fruit and vegetable consumption.
[c] Systolic and diastolic blood pressure, blood cholesterol, and heart rate.
[d] History of diabetes, coronary heart disease, self-reported stroke, arthritis, and respiratory diseases, and anti-depressant use at Phase 7.
[e] AH4-I test.
[f] Interleukin-6 and C-reactive protein (log-transformed).

Table S2 *Hazard ratios for cardiovascular and cancer mortality according to walking speed (bottom tertile versus middle and top tertiles combined).*

	**Cardiovascular mortality**			**Cancer mortality**		
**N deaths / N**	**62/6266**			**111/6266**		
**Adjustment**	**HR** [a]	**95% CI**	**% Δ**	**HR** [a]	**95% CI**	**% Δ**
Model 1: Adjusted for age and sex	2.24	1.34-3.74	--	1.62	1.11-2.36	--
Model 2: Model 1 + socioeconomic status	2.12	1.26-3.58	6.5	1.67	1.14-2.46	-7.3
Model 3: Model 1 + height + BMI	1.99	1.18-3.38	14.0	1.49	1.01-2.19	17.1
Model 4: Model 1 + health behaviours [b]	2.07	1.23-3.50	9.4	1.47	1.00-2.16	19.2
Model 5: Model 1 + cardiovascular risk factors [c]	2.19	1.31-3.66	2.8	1.59	1.08-2.32	3.9
Model 6: Model 1 + chronic conditions [d]	1.86	1.10-3.16	22.8	1.50	1.02-2.20	15.7
Model 7: Model 1 + cognitive function [e]	2.12	1.26-3.58	6.6	1.61	1.09-2.36	1.3
Model 8: Model 1 + inflammatory markers [f]	1.85	1.10-3.13	23.4	1.46	0.99-2.14	21.2
Model 9: Fully-adjusted	1.50	0.86-2.62	49.3	1.28	0.86-1.92	48.2

[a] HR computed using Cox proportional-hazards models with age as the time-axis for participants in the bottom tertile of walking speed (<1.26 m/s in men; <1.09 m/s in women) compared to participants in the top and middle tertiles combined. The assumption of proportionality of hazards was verified (cardiovascular, P=0.55; cancer, P=0.72). The percentage reduction (% Δ) of the association between walking speed and mortality attributed to covariates included in model *i* was calculated using the formula 100 x ($\beta_{Model\ 1}$ - $\beta_{Model\ i}$)/($\beta_{Model\ 1}$), where β is the regression coefficient obtained from the Cox proportional hazards model.
[b] Smoking history, alcohol consumption, physical activity, and fruit and vegetable consumption.
[c] Systolic and diastolic blood pressure, blood cholesterol, and heart rate.
[d] History of diabetes, coronary heart disease, self-reported stroke, arthritis, and respiratory diseases, and anti-depressant use at Phase 7.
[e] AH4-I test.
[f] Interleukin-6 and C-reactive protein (log-transformed).
